# Determining KLF14 tertiary structure and diagnostic significance in brain cancer progression

**DOI:** 10.1038/s41598-022-12072-0

**Published:** 2022-05-16

**Authors:** Kainat Zahra, Maria Shabbir, Yasmin Badshah, Janeen H. Trembley, Zunaira Badar, Khushbukhat Khan, Tayyaba Afsar, Ali Almajwal, Nawaf W. Alruwaili, Suhail Razak

**Affiliations:** 1 Department of Healthcare Biotechnology, Atta-ur-Rahman School of Applied Biosciences, National University of Sciences and Technology, Islamabad, Pakistan; 2 Department of Community Health Sciences, College of Applied Medical Sciences, King Saud University, Riyadh, Saudi Arabia; 3 Minneapolis VA Health Care System Research Service, Minneapolis, MN USA; 4 Department of Laboratory Medicine and Pathology, University of Minnesota, Minneapolis, MN USA; 5 Masonic Cancer Center, University of Minnesota, Minneapolis, MN USA

**Keywords:** Cancer, Molecular biology

## Abstract

Expression analysis of new protein targets may play a crucial role in the early detection and diagnosis of brain tumor progression. The study aimed to investigate the possible relation of KLF14, TPD52, miR-124, and PKCε in the development and progression of brain cancer and space occupying lesion (SOL) of the brain. One hundred human blood samples comprising varying diagnostic groups (SOL brain, grade I, II, III, IV) were analyzed by real-time quantitative PCR to determine the expression level of KLF14, TPD52, miR-124, and PKCε. TPD52 and PKCε were upregulated in brain cancer by 2.5- and 1.6-fold, respectively, whereas, KLF14 and miR-124 were downregulated in brain cancer. In metastatic and high-grade brain cancer, TPD52 and PKCε expression were up-regulated and KLF14 and miR-124 expression were down-regulated. Further, these genes were found to be differentially expressed in the blood of patients with SOL. Upregulation of TPD52 and PKCε, however, reduced expression of KLF14 and miR-124 in SOL of the brain as compared to healthy controls. Expression analysis of TPD52, KLF14, miR-124, and PKCε provided useful information on the differences existing between the normal brain and SOL, in addition to gliomas; thus, might prove to be useful having diagnostic or prognostic value.

## Introduction

Primary brain cancers comprise a collection of central nervous system (CNS) malignancies of various cellular origins with differing biological underpinnings^1^. CNS cancers have increasing incidence and considerable global morbidity and mortality^2,3^. Both environmental as well as genetic factors have been known to contribute to brain cancer and tumours^4,5^. Molecular studies have identified several proteins belonging to different pathways that participate in cancer progression and aggressiveness. The role of PI3K/Akt pathway and Ras/Raf/ERK pathway as key signalling cascades behind cancer development have been established through decades of experimentation^6–8^. Protein kinase C epsilon (PKCε), tumour protein D52 (TPD52), Krüppel-like factors 14 (KLF14) and miR-124 are molecular players that modulate signal transduction through these pathways and contribute to the progression, proliferation, and invasiveness of several cancers^9–12^.

KLF14 belongs to the family of transcription regulators, Kruppel-like factors (KLFs). KLF14 modulates several genes that are important for maintaining cell homeostasis. Loss of KLF14 is reported to enhance the centrosome amplification through PLK4 that leads to carcinogenicity^13^. The expression loss of other KLFs, KLF3 and KLF9, was previously reported in breast cancer and cervical cancer^14,15^. Down-regulation of the transcription factor KLF14 is observed in colorectal cancer as well as several other human malignancies such as breast, lymphatic, cervical, oral cavity, floor of mouth, and pancreas^16^. Its loss promotes centrosomes amplification and metabolic rewiring—a distinctive characteristic of carcinogenicity, prompting proposal of KLF14 as a tumour suppressor^13,16,17^. However, its expression analysis has not been performed in brain tumours so far; although, a study does indicate its significant role in neurological functioning associated with memory^18^.

Few studies have been conducted that report on the expression of TPD52, miR-124, and PKCε genes in brain cancer. Oncogene TPD52 expression is modulated by lncRNA FGD5-AS1 in glioblastoma^19^. Its elevated expression is found in many human cancers including breast cancer^20,21^, gastric cancer^22^, lung cancer^23^ and head and neck squamous cell carcinomas^24^. TPD52 is found to be a tumour marker in ovarian cancer and its expression is absent in benign tumours^25^. A tumour suppressive role for miR-124 is supported by many studies in brain cancer. MiR-124 targets components of STAT3, AURKA and Ras signalling in gliomas^26–28^, and its loss supports angiogenesis, stem cell-like traits and invasiveness^29^. Moreover, miR-124 demonstrated potential application as a diagnostic and prognostic tool for CNS disorders such as brain tumours and stroke^30^. MiR-124 is down regulated in oligodendroglioma^31^, human astroblastoma^32^ and GBM cell lines^33^, and a recent publication reported down-regulation of miR-124 in plasma of glioma patients^34^. PKCε oncogenic contribution in numerous cancers is reported. PKCε causes cancer cell proliferation, apoptosis evasion, angiogenesis and drug resistance^35–37^. Up-regulated PKCε expression is demonstrated in astrocytoma tumor^38^, but investigation on PKCε expression analysis in human tissue for other brain cancers is lacking.

Although the expression of miR-124 in brain cancer is extensively studied, its expression in cancer patient blood is rarely reported. Studies delineating TPD52, KLF14 and PKCε expression in brain tumours and space occupying lesions (SOL) of brain are also scarce. Literature review indicated that the tertiary structure of KLF14 is not established so far. Knowledge of protein structure is extremely important in gaining in-depth insight of protein’s molecular interactions. Such information presents value in targeted drug design and better understanding of target protein molecular interactions. Thus, in the present study we aimed to predict KLF14 three-dimensional structure to contribute information about its function and differences that may exist between normal brain, SOL of brain and gliomas.

SOL presents a large dilemma in diagnosis of brain cancers. Late diagnosis of neoplasm SOL leads to neurological damage and behavioural change^39,40^. Several studies have been dedicated to diagnosing neoplasm SOL at early stages^41,42^. Given the heterogeneous nature of brain cancer and SOL, effective diagnostic and treatment strategies require delineation of co-expression profiles for multiple genes. Therefore, the main aim of this study was to investigate the gene expression levels and possible correlation between PKCε, TPD52, miR-124 and KLF14 to improve understanding of their contribution in the development of brain cancer and neoplasm lesions. A potential advantage of this blood-based analysis toward cancer diagnosis or prognosis is that acquisition of brain biopsy samples in not always possible^43^.

## Material and methods

### Blood sample collection

Inclusion criteria included biopsy-, chemotherapy- or radiotherapy- diagnosis of brain tumour. Study subject ages ranged between 20 and 60 years. Exclusion criteria included other simultaneous diseases such as heart disease, diabetes, and hypertension. Subjects were asked to sign a consent form for participation in the study. Ethical approval was acquired from institutional review board of Atta-ur-Rahman School of Applied Biosciences, NUST and Combined Military Hospital (CMH), Rawalpindi. All participants were informed about the study objectives and signed informed consent. The study protocol was carried out in accordance to the principles of the Declaration of Helsinki^44^.

Blood samples (3 mL) from 80 brain cancer and 20 SOL of brain patients were collected. HTS sterile tubes were used for the collection of blood and instantly put on ice to avoid RNA degradation. Samples belonged to different subtypes of gliomas, as indicated in Table 1. Clinical and pathological features, including age, cancer grade, metastasis, and gender, are detailed in Table 2. All patients were on standard treatment, according to U.S. Food and Drug Administration clinical guidelines. For controls, blood samples were collected from 50 females and 50 males (age 20 to 60) with no documented comorbidity.

**Table 1:** No of cases observed in the study according to their clinical and pathological feature. SOL Brain.

Clinical features	No of patients N (%)
Age	
> 50	68(68)
< 50	32(32)
Primary tumors	60(75)
Secondary tumors	20(25)
Grade	
I	
Pilocytic astrocytoma	5(5)
II	
Astrocytoma	6(6)
Oligodendroglioma	10(10)
III	
Astrocytoma	4(4)
Oligodendroglioma	5(5)
IV	
Glioblastoma multiform	30(30)
Metastatic cancer	20(20)
SOL brain	20
Gender	
Male	64(64)
Female	36(36)

**Table 2:** No of cases observed in the study according to their clinical and pathological feature.

Clinical features	No of patients N (%)
Age	
> 50	68(68)
< 50	32(32)
Metastatic	
Non-metastatic	80(80)
Metastatic	20(20)
Stage	
I + II	50(50)
III + IV	50(50)
SOL brain	20
Gender	
Male	64(64)
Female	36(36)

### RNA extraction and cDNA synthesis

Total RNA extraction was conducted from whole blood using TriZol reagent^45^. The samples were kept on ice during the whole procedure to avoid degradation. NanoDrop 2000 (Thermo Fisher Scientific, Waltham, MA, USA) was used to determine the concentration and purity of RNA. Samples with A260/A280 > 1.7 ratio were used for cDNA synthesis. For cDNA synthesis, 20 µL reactions were prepared by adding 1000 ng (1 µl) of extracted RNA, 1 µl of oligo dT20 primers and 1 µl dNTP mix (2.5 mM) (add name of cDNA kit and manufacturer). Reactions proceeded at 65 °C for 5 min in a thermocycler, followed by incubation on ice. In the next step, 2 µl of the 10X reaction buffer, 1 µl of DTT (100 Mm), 0.5 µl RNase inhibitor and 1 µl RTase were added. Then it was further processed in the thermocycler at 42 °C for 50 min and then at 70 °C for 10 min. The cDNA was stored at −20 °C.

### Expression analysis of TPD52, PKCε, KLF14 and miR-124

The expression of TPD52, PKCε, KLF14 and miR-124 was carried out by using real time PCR analysis. Primer used for expression analysis of TPD52 forward 5′-GCTGCTTTTCGPCTGTTGGCT-3′, reverse 5′-TCAAATGATTTAAAAGTTGGGGAGTT-3′, PKCε forward 5′-AGCCTCGTTCACGGTTCT-3′, reverse 5′-TGTCCAGCCATCATCTCG-3′, KLF14 forward 5′-CCACCCAACCTATCATCCAG-3′, reverse 5′-GTACCTCCCCAGAGTCCACA-3` and miR-124 forward 5′-GATACTCATAAGGCACGCGG-3′, reverse 5′-GTGCAGGGTCCGAGGT-3′. WizPure qPCR master mix (SYBR) was used to prepare the reaction mixture (manufacturer info). The reaction mixture comprised of 10 µl of SYBR, 0.4 µl of forward and reverse primers, 2 µl of cDNA and 6.8 µl of nuclease free water. The thermocycler conditions were as follows: initial denaturation at 95 °C for 10 min, followed by 40 cycles of denaturation at 95 °C for 30 s, annealing at 60 °C for 30 s and extension at 72 °C for 30 s. All samples were run in triplicate. For normalization, GAPDH primers were used (forward 5’GTCTCCTCTGACTTCAACAGCG3’ and reverse 5’ACCACCCTGTTGCTGTAGCCAA3″). 2-▲▲CT method was used for the gene expression quantification.

### Statistical analysis

GraphPad Prism software version 8.0.1 was used for performing statistical analysis. Fold change was calculated using Ct values. ANOVA 9 (multiple comparisons) was used, and results were considered statistically significant when *P*-value was less than 0.05. ROC curve analysis was employed via Graphpad prism and Area under the curve (AUC) along with 95% confidence interval score was determined.

### Construction of 3D structure for KLF14

3D structure of KLF14 was determined by using an insilico approach. The amino acid sequence of KLF14 (gene ID: 136,259) was retrieved from NCBI. The sequence used was in FASTA format. The subcellular localization of KLF14 was predicted using DeepLoc-1.0^46^, Hum-mPLoc 3.0^47^ and PSORT^48^. For multiple sequence alignment, all amino acid sequences of KLF family (1–16) were collected from NCBI in FASTA format and aligned by using “ClustalW”^49^. The Aligned sequences were analysed to find out the conserved domains of all KLF members. Web-based tools TMHMM 2.0^50^, TmPred 2.0^51^ and HHpred^52^ were used to predict transmembrane domains of KLF14. Various tools such as Spider 2^53^, PSIPRED^54^, I-TASSER^55^ and PSSPRED were employed to predict the secondary structure of KLF14. Phylogenetic analysis of the KLF proteins were performed using Mega X^56^. KLF4 structure was used as a template for homology modelling. The crystal structure of KLF4 (2wbu) was retrieved from Protein Data Bank RCSB^57^. Homology modelling server of Swiss-Model Workspace^58^ was used to construct the 3D structure. Three dimensional structures were envisaged by using Chimera^59^.

### Pathway construction

To construct a cellular pathway and establish a crosstalk between understudied genes, KEGG and String analyses were performed, and the pathway was built via DAVID software.

## Ethics approval and consent to participate

The experimental protocol for the use of Human was approved (Ref: No: IRB-110) by the ethical committee of Combined Military Hospital and ASAB, NUST. Informed consent was taken from all participants of the study.

## Results

### Expression of TPD52, PKCε, KLF14 and miRNA-124 in blood of brain cancer patients

**Figure 1:**
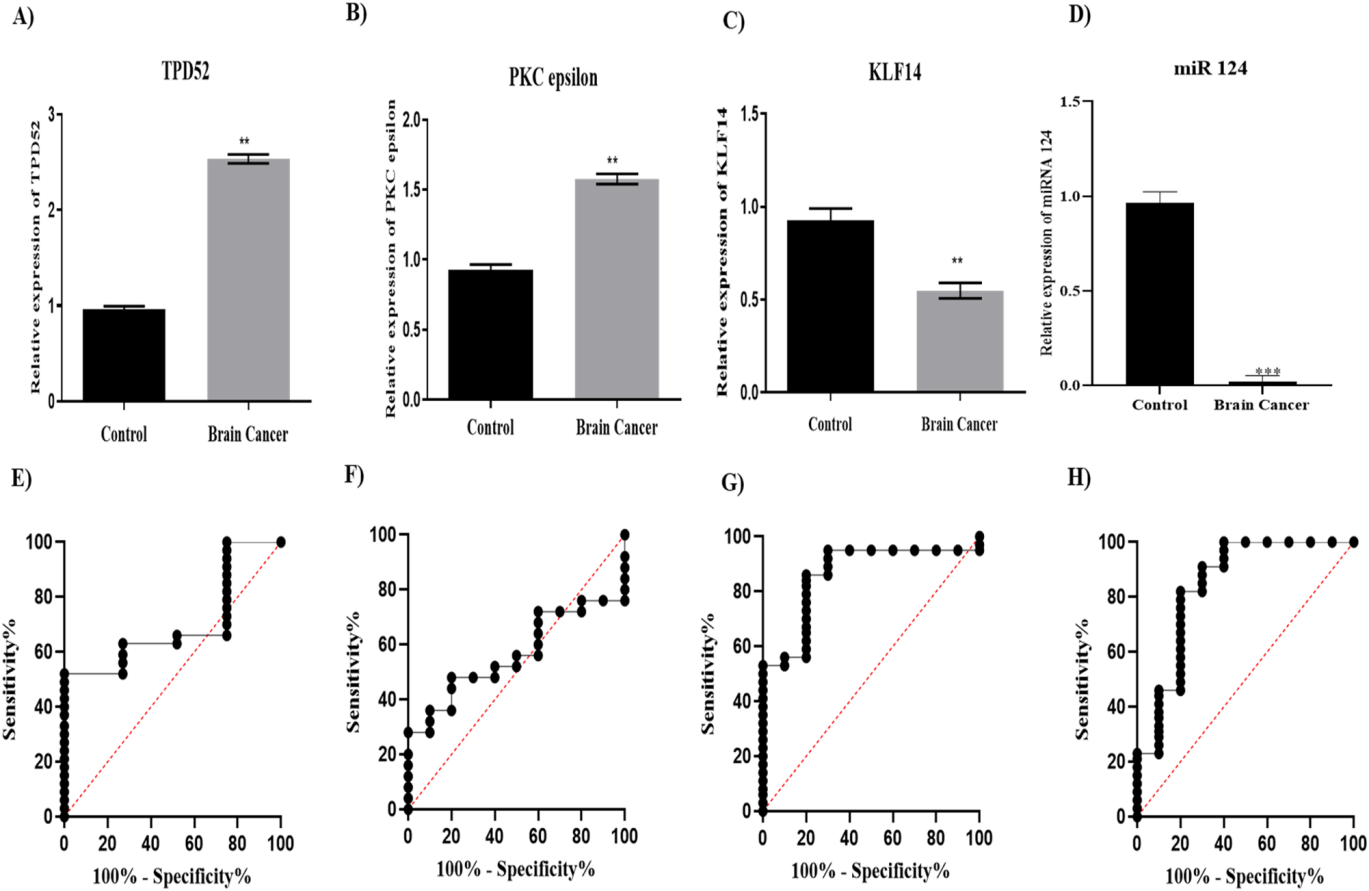
Expression and diagnostic significance of TPD52, PKCε, KLF14 and miRNA 124. Expression of (**A**) TPD52 (**B**) PKCε (**C**) KLF14 and (**D**) miR-124 in brain tumor patients compared to healthy controls. There is up-regulated expression of TPD52, and PKCε (*P* = 0.0015, *P* = 0.0046 respectively) and down-regulated expression of KLF14 and miRNA124 (*P* = 0.0025, *P* = 0.0010 respectively) in brain tumor patients compared to healthy controls. Representative data were presented as mean ± SEM of triplicate experiments and **refers to *P* value less than 0.05 and ***refers to *P* value less than or equal to 0.001. Statistical significance was measured by two-way ANOVA. ROC curve for TPD52, PKCε, KLF14 and miR-124 predicted high risk for brain tumor. Area under the ROC curve (AUC) for (**E**) TPD 52 was 0.69 and 95% confidence interval (CI) was 0.6236 to 0.7758. AUC for (**F**) PKCε was 0.564 and 95% CI was 0.4804 to 0.6476. AUC for (**G**) KLF14 was 0.86 and 95% CI was 0.8057 to 0.9143. AUC for (**H**) miR-124 was 0.8420 and 95% CI was 0.7858 to 0.8982.

This study utilized 80 patient blood samples from different brain cancer subtypes, including astrocytoma, oligodendroglioma and glioblastoma and 100 blood samples from healthy control individuals. Gene expression analysis of the brain cancer samples by qRT-PCR revealed that there was 2.5-fold higher expression of TPD52 in blood of brain cancer patients as compared to healthy controls (Fig. 1A, *P* = 0.0015). Elevated PKCε expression (1.5-fold) was also observed in cancer patients as compared to healthy controls (Fig. 1B, *P *= 0.0046). KLF14 mRNA levels were 50% lower in cancer patients as compared to healthy controls (Fig. 1C, *P* = 0.0025). A striking reduction in miR-124 level was observed in the blood of brain cancer patients with expression decreased to 0.002 of healthy controls (Fig. 1D, *P* = 0.001).

Diagnostic significance of the expression of studied genes in brain tumor was determined through receiver operating characteristic (ROC) curve analysis (Fig. 1E–H). The analysis revealed that the value for KLF14 and miR-124 in predicting risk of brain tumor is high with Area under the Curve (AUC) 0.86 and 0.84, respectively. The value for TPD52 and PKCε also depicted their significance in predicting the brain tumor risk (AUC 0.62 and 0.56, respectively).

### TPD52, PKCε, KLF14 and miRNA-124 expression with tumour grade, metastasis in brain cancer

**Figure 2:**
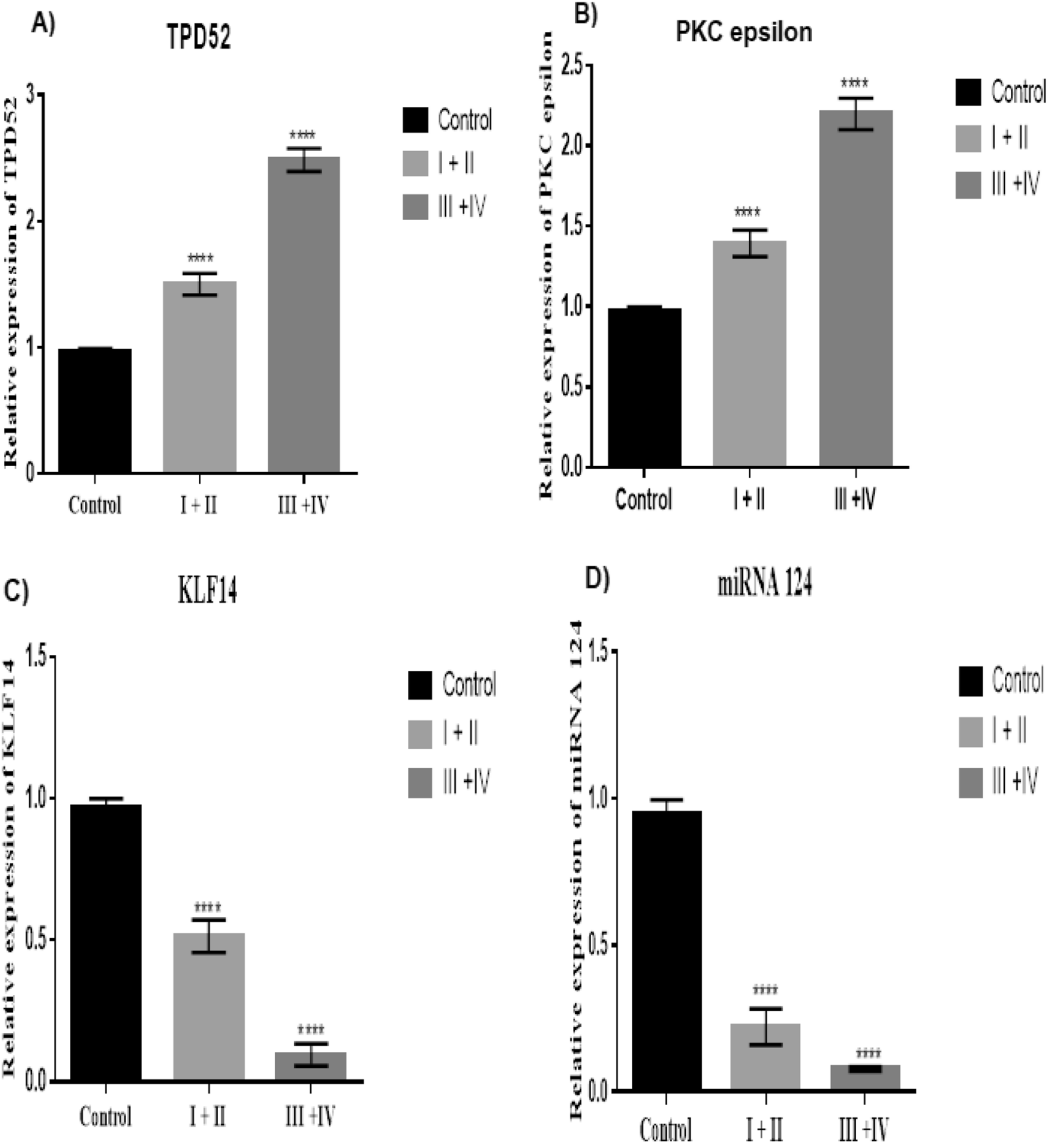
Expression of TPD52, PKCε, KLF14 and miRNA124 in relation to tumor grade of brain cancer. (**A**) Expression of TPD52 in grade I + II and III + IV compared to healthy controls; (**B**) Expression of PKCε in grade I + II and III + IV patients compared to healthy controls; (**C**) Expression of KLF14 grade I + II and III + IV patients compared to healthy controls; (**D**) Expression of miRNA 124 in grade I + II and III + IV compared to healthy controls; Representative data are presented as mean ± SEM of triplicate experiments. Statistical significance was measured by one way Anova (****P < 0.0001).

Evaluation of gene expression levels for TPD52, miR-124, PKCε and KLF14 with respect to cancer grade was performed (Table 2). These genes showed an association with tumour grade (*P* < 0.0001). In the advanced grade group, there was higher expression of TPD52 and PKCε as compared to lower grade group (Fig. 2A, B, respectively). KLF14 and miR-124 showed low expression in advanced grade groups as compared to lower grade groups (Fig. 2C, D respectively).

**Figure 3:**
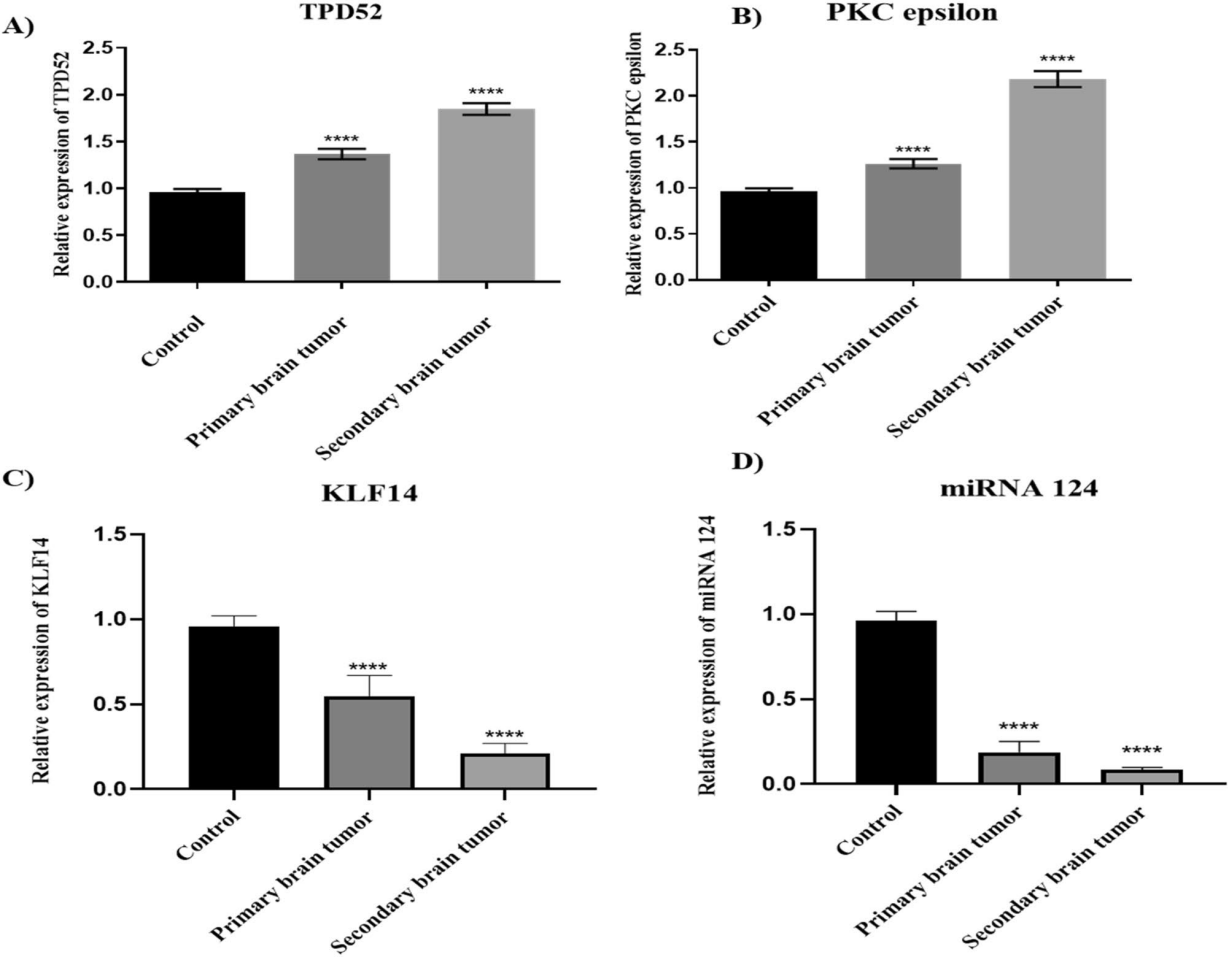
Expression of TPD52, PKCε, KLF14 and miRNA124 in primary and secondary groups of brain cancer. (**A**) Expression of TPD52 in metastatic and non-metastatic groups of brain cancer patients compared to healthy controls; (**B**) Expression of PKCε in metastatic and non-metastatic groups of brain cancer patients compared to healthy controls; (**C**) Expression of KLF14 in metastatic and non-metastatic groups of brain cancer patients compared to healthy controls; (**D**) Expression of miRNA 124 in metastatic and non-metastatic groups of brain cancer patients compared to healthy controls. There is an elevated expression of TPD52 and PKCε in metastatic group compared to non-metastatic group (*P* < 0.0001). KLF14 and miRNA124 are low in expression in metastatic group compared to non-metastatic group (*P* = 0.0005, *P* < 0.0001 respectively). Representative data were presented as mean ± SEM of triplicate experiments.). Statistical significance was measured by one-way ANOVA (****P < 0.0001 and ***P < 0.05).

Differential expression of these understudied genes in relation to primary and secondary metastatic status of brain cancer was also investigated (Table 2). TPD52 and PKCε were observed to be highly expressed in the secondary metastatic group as compared to the primary metastatic group of brain cancer patients (*P* < 0.0001) (Fig. 3A and Brespectively). Lower expression of KLF14 (Fig. 3C) and miR-124 (Fig. 3D) was observed in the secondary metastatic group compared to the primary metastatic group (*P* = 0.0001, *P* < 0.0001 respectively).

**Figure 4:**
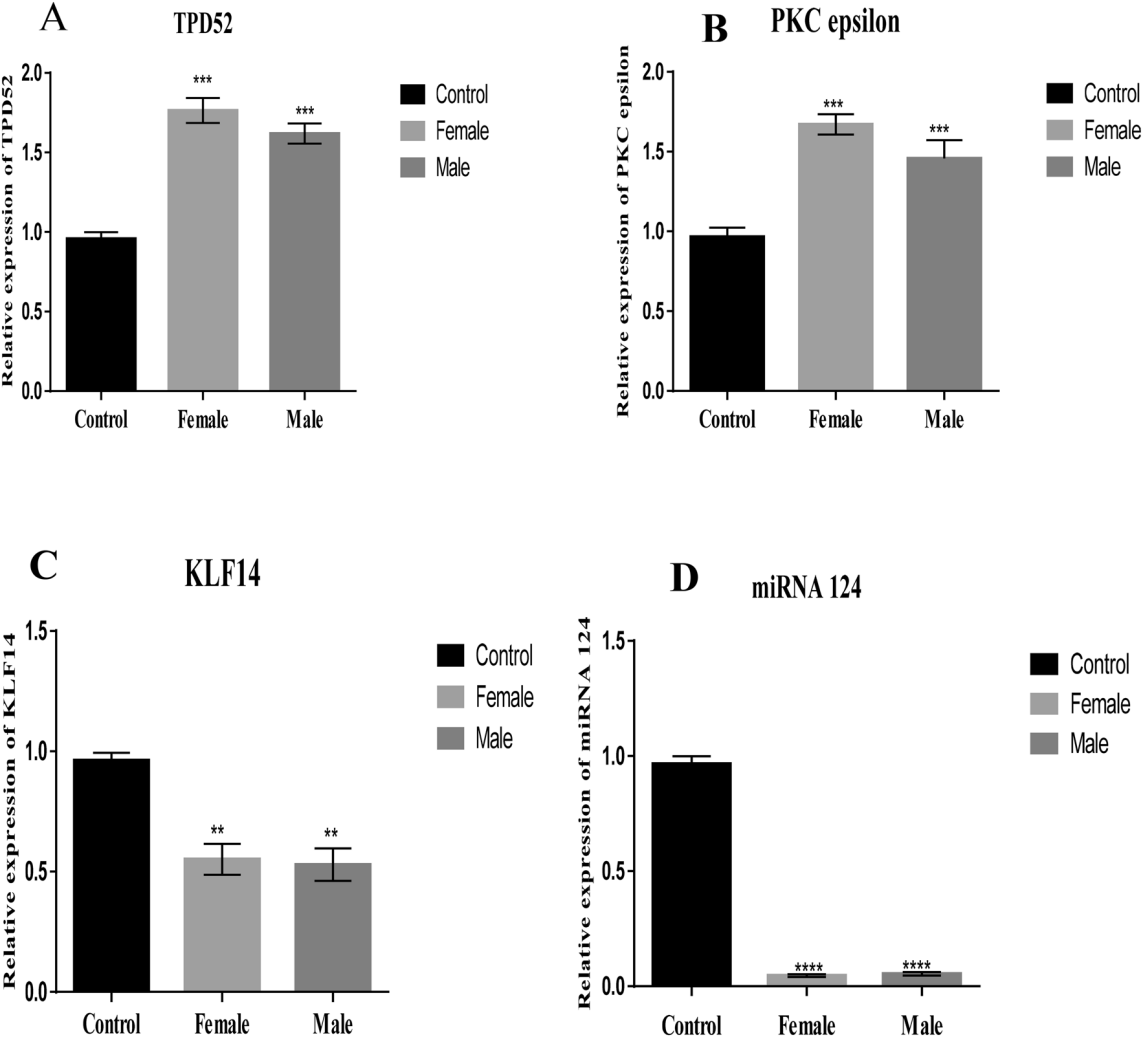
Expression of TPD52, PKCε, KLF14 and miRNA124 in male and female patients. (**A**) Expression of TPD52 in male and female patients compared to healthy male and female controls, respectively; (**B**) Expression of PKCε in male and female patients compared to healthy male and female controls, respectively; (**C**) Expression of KLF14 in male and female patients compared to healthy male and female controls, respectively; (**D**) Expression of miRNA 124 in male and female patients compared to healthy male and female control, respectivelys. There is significant difference of PKCε, TPD52, miRNA124, and KLF14 between male and female patients group and healthy control (*P* = 0.0003, *P* = 0.0002, *P* < 0.0001, *P* = 0.0027 respectively). Representative data were presented as mean ± SEM of triplicate experiments. Statistical significance was measured by one-way ANOVA (****P < 0.0001, ***P < 0.01, **P < 0.05).

TPD52, miRNA-124, PKCε and KLF14 were evaluated for altered expression levels separately in male and female patient groups (Table 3). The expression of TPD52 and PKCε was higher while miR-124 and KLF14 was lower in both males and females in comparison to the control group (Fig. 4). However, the expression of TPD52 and PKCε were up-regulated in females in comparison to males (*P* = 0.0002 and *P* = 0.0003, respectively). KLF14 expression (*P* = 0.0027) was comparatively lower in males while miR-124 (*P* < 0.0001) expression was comparatively lower in females.

**Table 3:** Expression of TPD52, PKC epsilon, KLF14 and miR-124 across the clinical features in brain tumors. The table shows the fold change and *P* values.

Clinical features	Expression of TPD52 (Fold change)	*P* value	Expression of PKCε (Fold change)	*P* value	Expression of KLF14 (Fold change)	*P* value	Expression of miR-124 (Fold change)	*P* value
**Grade**		< 0.0001		< 0.0001		< 0.0001		< 0.0001
I + II	1.490926		1.371742		0.412434		0.178404	
II + III	2.42522		2.012914		0.112212		0.068463	
**Metastasis**		< 0.0001		< 0.0001		= 0.0001		< 0.0001
Metastatic	1.735585		2.041288		0.145966		0.099246	
Non-metastatic	1.384441		1.2526		0.420548		0.155094	
**SOL brain**		< 0.0001		< 0.0001		< 0.0001		< 0.0001
Cancer patients	2.230162		2.142817		0.046102		0.018919	
SOL patients	1.280062		1.81003		0.455714		0.167241	
**Gender**		= 0.0002		= 0.0003		= 0.0027		< 0.0001
Male	1.506463		1.334528		0.51136		0.052079	
Female	1.892561		1.770301		0.676788		0.048701

### Expression of TPD52, PKCε, KLF14 and miRNA-124 in space occupying lesion (SOL) of the brain

**Figure 5:**
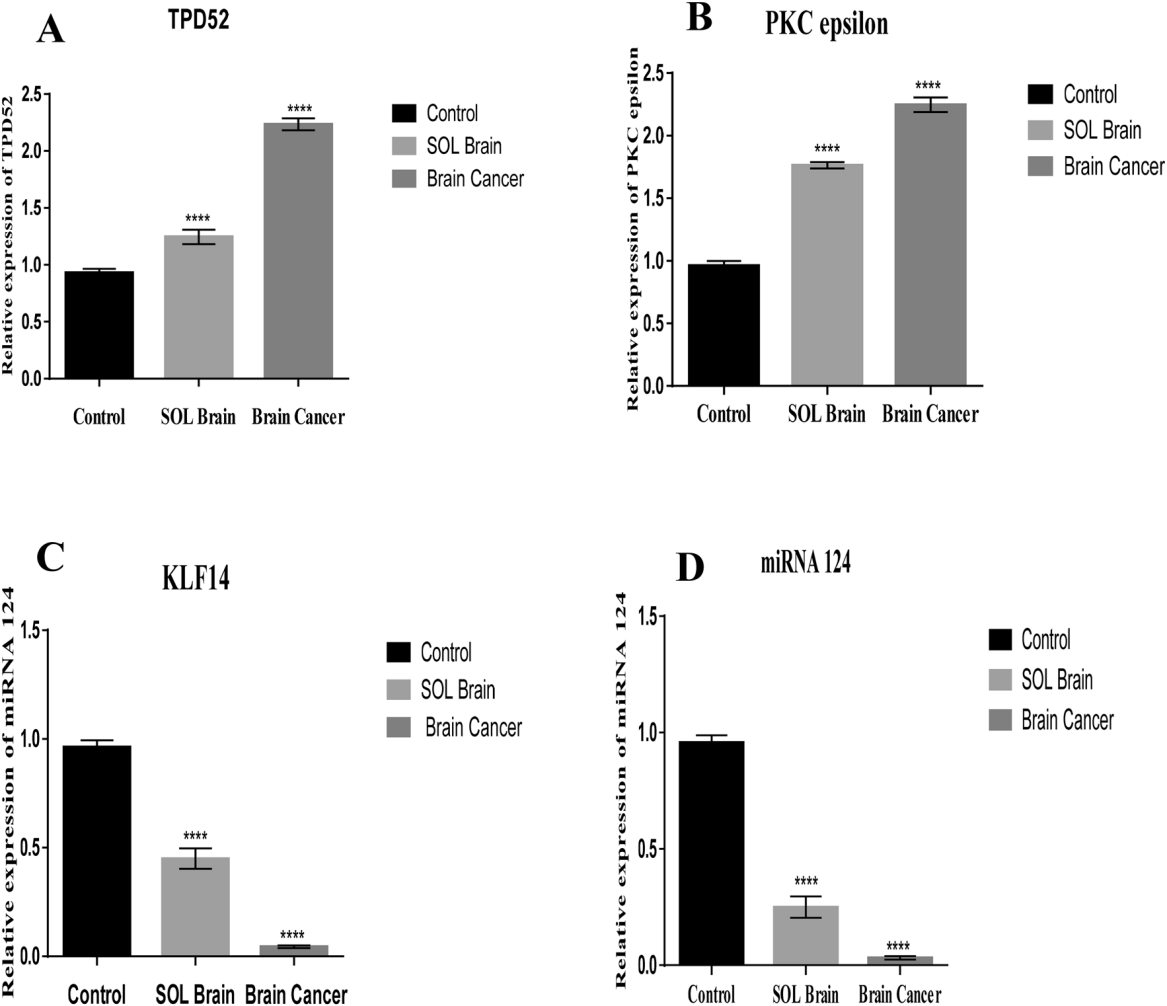
Expression of TPD52, PKCε, KLF14 and miRN-124 in SOL. (**A**) Expression of TPD52 in SOL brain compared to healthy controls and cancer patients; (**B**) Expression of PKCε in SOL brain patients compared to healthy controls and cancer patients; (**C**) Expression of KLF14 in SOL brain compared to healthy controls and cancer patients; (**D**) Expression of miRNA 124 in SOL brain compared to healthy controls and cancer patients. Representative data are presented as mean ± SEM of triplicate experiments. Statistical significance was measured by one-way ANOVA (****P < 0.0001).

Twenty blood samples from patients with space occupying lesions (SOL) of brain were collected and analysed for expression of TPD52, miR-124 PKCε and KLF14 (Table 3). These genes were found to be differentially expressed. There was elevated expression of TPD52 and PKCε compared to healthy control (*p* < 0.0001). The expression of TPD52 and PKCε was lower in SOL patients in comparison to patients with high grade cancer (Fig. 5A, B, respectively). KLF14 and miR-124 mRNA levels were decreased in SOL samples relative to healthy control (*p* < 0.0001), but to a lesser extent than the reductions in high grade cancer (Fig. 5C, D, respectively).

### Expression of TPD52, PKCε, KLF14 and miRNA 124 in astrocytoma, glioblastomas, and oligodendrogliomas

**Figure 6:**
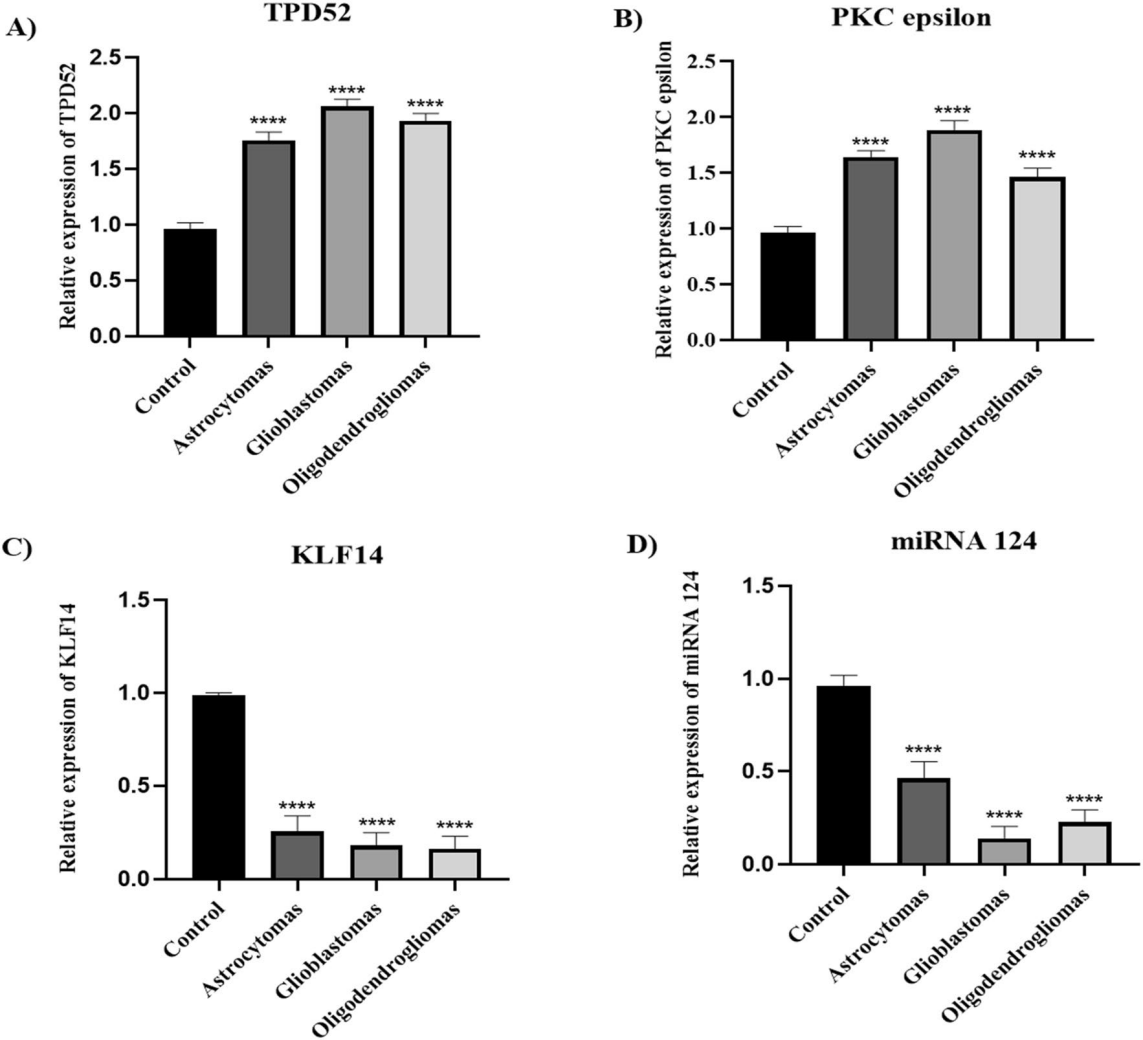
Expression of TPD52, PKCε, KLF14 and miRNA124 in brain tumor subtypes. (**A**) Expression of TPD52 in astrocytomas, glioblastomas, and oligodendrogliomas patients compared to healthy controls; (**B**) Expression of PKCε in astrocytomas, glioblastomas, and oligodendrogliomas patients compared to healthy controls; (**C**) Expression of KLF14 in astrocytomas, glioblastomas, and oligodendrogliomas patients compared to healthy controls; (**D**) Expression of miRNA 124 in astrocytomas, glioblastomas, and oligodendrogliomas patients compared to healthy controls**.** There is an elevated expression of TPD52 and PKCε in glioblastoma compared to astrocytomas and oligodendrogliomas. KLF14 expression was lower in glioblastomas, and oligodendrogliomas in comparison to astrocytomas. miRNA 124 expression was higher in astocytomas in comparison to glioblastomas, and oligodendrogliomas but lower compared to healthy control. Representative data were presented as mean ± SEM of triplicate experiments. Statistical significance was measured by one-way ANOVA (****P < 0.0001).

Differential expression analysis of TPD52, PKCε, KLF14 and miRNA 124 in brain cancer subtypes: astrocytomas, glioblastomas, and oligodendrogliomas revealed that the expression of TPD52 and PKCε was up regulated in glioblastomas compared to astrocytomas and oligodendrogliomas (Fig. 6A and B). However, TPD52 expression was lower comparative to glioblastomas, and oligodendrogliomas but significantly up-regulated relative to healthy control. In comparison to astrocytomas and glioblastomas, the expression of PKCε was lower in oligodendrogliomas but elevate comparative to healthy individual. The expression of KLF14 and miR-124 was downregulated when compared to control. However, KLF14 expression was higher in astrocytomas relative to glioblastomas, and oligodendrogliomas (Fig. 6C) while expression of miR-124 was higher in astrocytomas and oligodendrogliomas compared to glioblastomas (Fig. 6D).

### Identification of pathways instituting crosstalk between TPD52, KLF 14, PKCε, and miR-124

**Figure 7:**
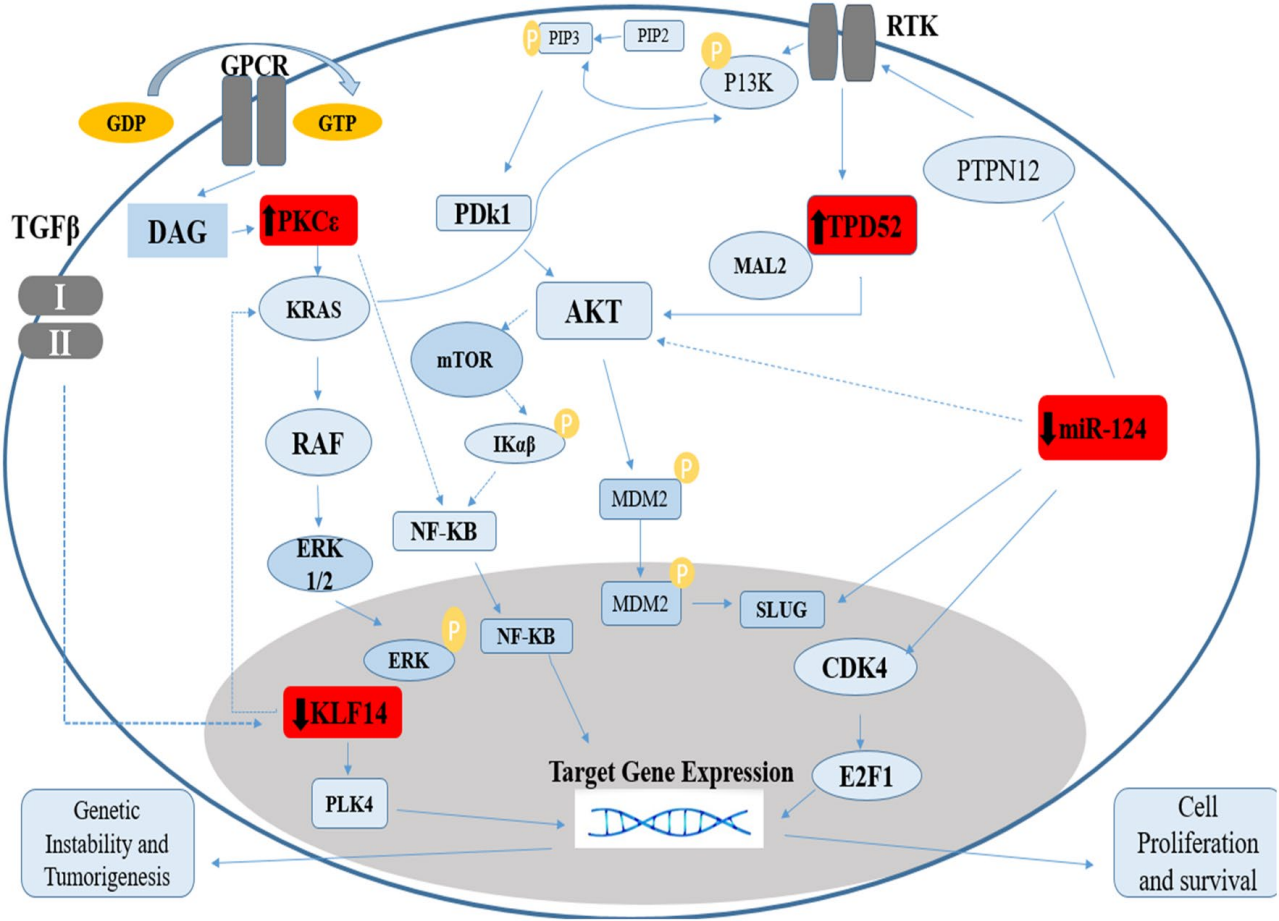
The involvement of Akt and KRas pathways in brain cancer. Akt and KRas pathways are majorly involved in progression of cancer. These two pathways are influenced by PKCε, TPD52, miR-124 and KLF14, in addition to several other downstream effectors. The deregulated expression of TPD52, KLF14 and miR-124 and PKCε contribute to the over-expression of these two oncogenic pathways which in turn promotes cell survival and proliferation.

Several analysis tools were employed to determine potential pathways involving our genes of interest TPD52, PKCε, KLF14 and miR-124. Using KEGG and String, the output data indicated that these genes are linked with each other and are involved in the Akt pathway. Further pathway information obtained via DAVID software showed that PKCε is found upstream of the Ras/Raf pathway and regulates signal transduction from G-Protein-Coupled Receptor (GPCR) to KRas. A summary compilation of these analyses is shown in Fig. 7.

### Multiple sequence alignment including KLF14

**Figure 8:**
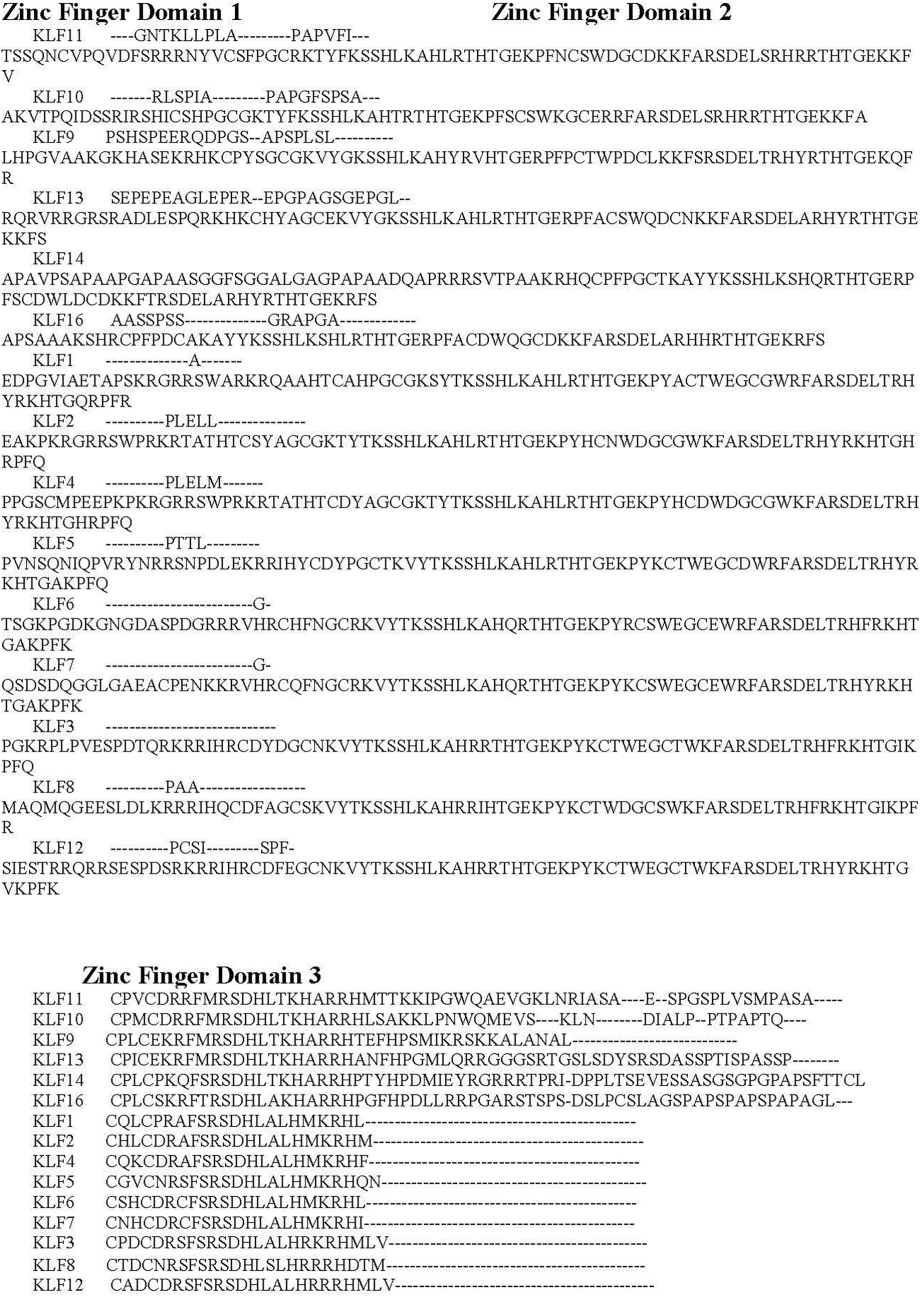
Sequence alignment of Kruppel like factors depicting conserved domains obtained from Clustal Omega. Certain sequence alignments have been deleted for formatting, Zinc figure domain 1(labeled as green), zinc figure domain 2 (labeled as purple) and zinc figure domain 3 (labeled as blue) have been highlighted.

To provide further understanding of KLF14, multiple sequence alignments were carried out among the members of the KLF family. Figure 8 shows the Clustal Omega sequence alignment of the fifteen Kruppel-like factors, which depicts the three conserved Zinc finger domains across the KLF family. The Zinc finger domain is the most important feature of Kruppel-like factors. It contains three classical Cys2-His2 zinc fingers and is observed to be conserved in all the known KLFs. Moreover, alignment of amino acid sequences of the zinc fingers reflects a high degree of sequence identities. These zinc finger domains interact with CACCCC elements and GC-rich regions of DNA to initiate activation and repression of transcription^60^.

### Phylogenetic analysis

**Figure 9:**
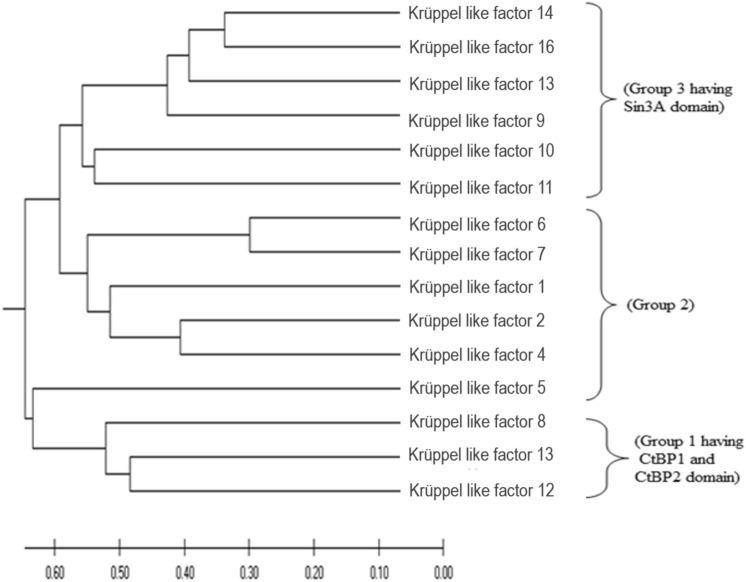
Phylogenetic analysis of KLF family. The evolutionary history was inferred using the UPGMA method. The optimal tree with the sum of branch length = 7.54632578 is shown. The tree is drawn to scale, with branch lengths in the same units as those of the evolutionary distances used to infer the phylogenetic tree. The evolutionary distances were computed using the Poisson correction method and are in the units of the number of amino acid substitutions per site. This analysis involved 15 amino acid sequences. All ambiguous positions were removed for each sequence pair (pairwise deletion option). There was a total of 602 positions in the final dataset. Evolutionary analyses were conducted in MEGA X^42^.

Phylogenetic deduction is an important aspect in functionally analysing a gene family. Full-length amino acid sequences encoding KLF proteins were used to construct UPGMA tree (Fig. 9) by Mega X^56^. Two large groups with respect to phylogenetic clades were clearly visible in the tree, containing further subgroups. KLFs are divided into three subgroups; Group 1 (KLF 3, 8, and 12) are repressors that associates with C-terminal Binding Protein 1 and 2 (CtBP1 and CtBP2), Group 2 (KLFs 1, 2, 4, 5, 6, and 7) are transcription activators, Group 3 (KLFs 9, 10, 11, 13, 14, and 16) shows repressor activity by interacting with the common transcriptional co-repressor, Sin3A^61^. Further, CtBP domains and Sin3A-binding domains are conserved in most eukaryotic species^62^.

### 3D structure of KLF14

**Figure 10:**
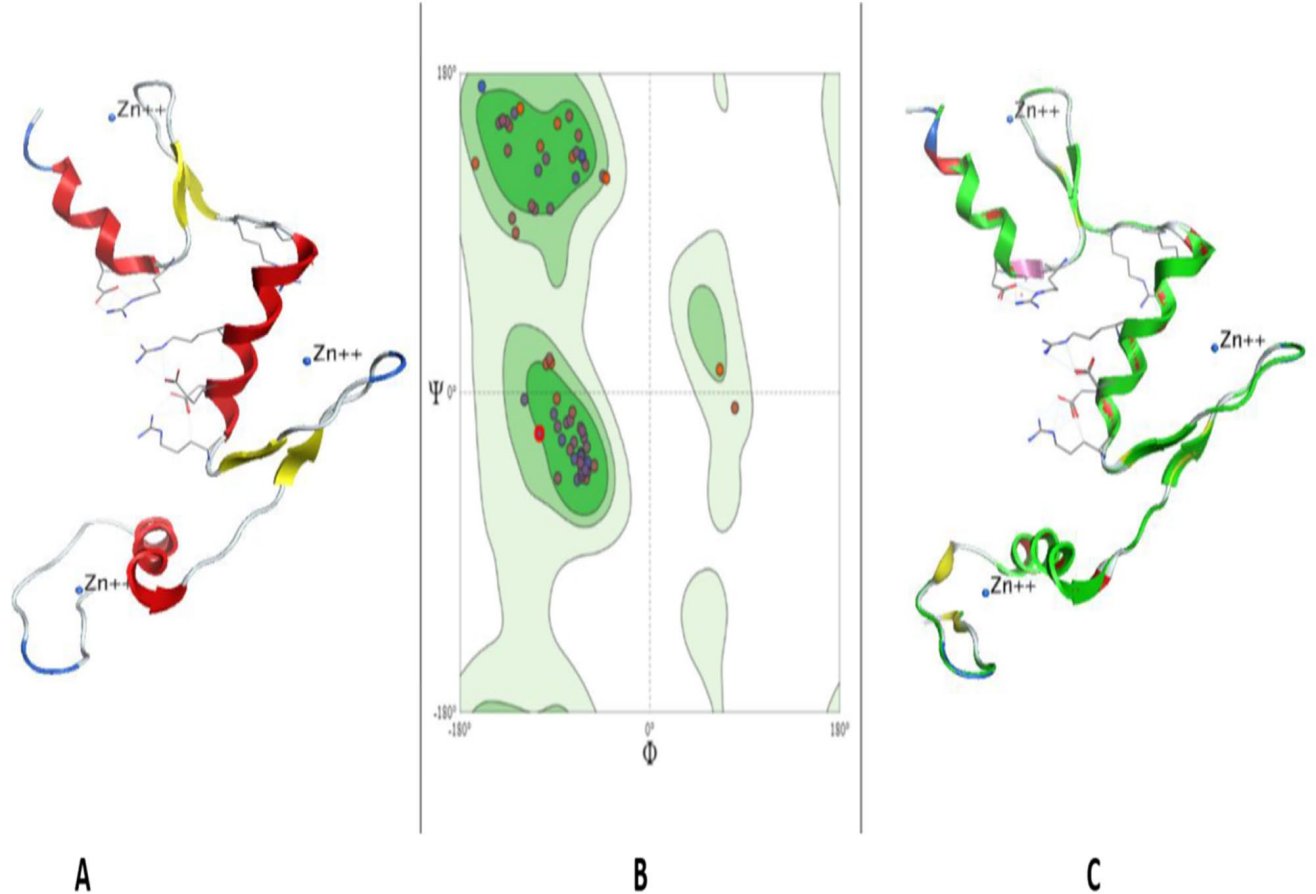
Tertiary structure prediction of KLF14 through homology modelling. (**A**) 3D structure of KLF14—Alpha helices, beta sheets, coils and three zinc ions are shown. (**B**) Ramachandran plot examining the quality of the model. The majority of the amino acids are traced out in the favoured region exhibiting that the model is of good stereochemical quality. (**C**) Superimposed structure of KLF14 (green) and KLF4 as template (red). KLF14 shows matching with alpha helices of template protein chain and a Q-Score of 0.976.

Structure of KLF14 (NCBI ID: NP_619638.2) (Fig. 10A) obtained by Swiss model was found to be 95.06% favoured by Ramachandran plot (Fig. 10B). The three-dimensional structure was constructed by using KLF4 (protein id: 2wbu) as a template retrieved from RCSB PDB that undertakes 61.45% sequence identity of KLF14 with KLF4. Three-dimensional (3D) structure of KLF14 (Fig. 10A) has three zinc-finger domains near the C-terminus, all three are of classical C2H2 type. The dihedral angles (phi against psi) of amino acids that have particular role in secondary structure are visualized by Ramachandran plot (Fig. 10B). MolProbity score was 1.23 and Ramachandran Outliers were 2.47%. However, some bad angles and bonds are formed as a result of derangements. In total, 1/719 bad bond and 19/965 bad angles were identified in the structure. In the upper right side, loops and helices are allowed, however, the upper left region favours beta sheets. The lower left region favours alpha helices, and left lower part disfavours the protein structure (Fig. 10B).

Superimposition results (Fig. 10C) revealed 61.45% identity between the constructed structure of KLF14 (green) and the template KLF4 (red).

### Uniqueness of KLF14

**Figure 11:**
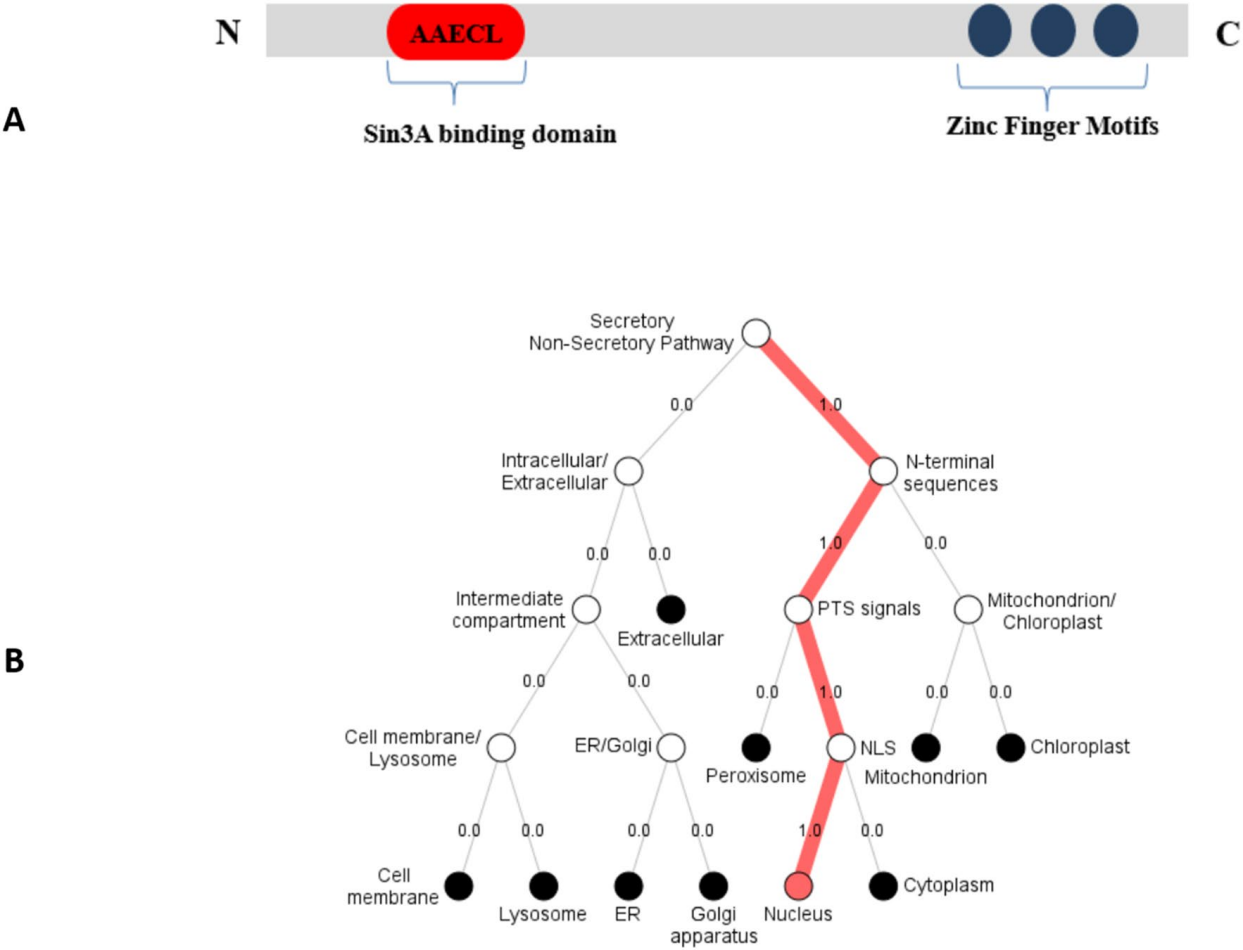
KLF14’s domains and cellular localization. (**A**) Primary structure of KLF14 showing the uniqueness of protein. Three zinc finger domains at C terminus and Sin3A binding domain at N terminus. (**B**) Localization of KLF14. DeepLOc 1.0^32^ shows strong evidence of the presence of KLF14 inside nucleus.

The primary structure of KLF14 (Fig. 11A) shows the three zinc finger domains at the C terminus and a Sin3A binding domain at the N terminus. The conserved Sin3A binding motif (AAECL, amino acids 12–16) shows repressor activity when bound with the Sin3A transcription factor^61^. Moreover, analysis using the Spider2 package revealed the alpha-helical conformation of this motif. Primary structure of KLF14 (Fig. 10A) shows the three zinc finger domains at C terminus and Sin3A binding domain at N terminus. Sin3A binding domain has a specific amino acid sequence of AAECL. KLF13 interacts with Sin3A through AAECL domain and act as transcription repressor.

### Localization of KLF14

Intracellular localization was determined using DeepLoc 1.0 that predicted KLF14 cellular localization inside the nucleus. This prediction supports KLF14 function as transcriptional regulator (Fig. 11B).

## Discussion

Brain cancer has been classified as the fatal cancer type that has relatively shorter survival rate Its increasing incidence has also become a source of concern^63,64^. Gene expression dysregulation plays an important role in the development of brain tumours. For example, elevated risk of sub ependymal giant cell astrocytoma is brought about due to a single gene disorder associated to the “*p*” arm of chromosome 9^56^. Therefore, it is necessary to investigate genes that may be involved in the progression of brain tumours in order to identify targets for treatment and diagnosis of the disease. The present study evaluated differential expression of four genes: PKCε, TPD52, KLF14 and miR-124 that are involved in modulation of Akt/PI3K and Ras/Raf/ERK1/2 signaling in different cancers.

Previously, co-expression of PKCε, TPD52, and miR-124 with KLF3 was reported in breast cancer^36^. PKCε down-regulation at the transcriptomics level indicated its tumor-suppressive function in breast cancer. In the current study, elevated PKCε expression was found. Expression of miR-124 and TPD52 were shown to be decreased and increased, respectively, in blood of breast cancer patients, a result that is in accordance with the findings of the current study.

KLF14 has an important role in brain functions, including cell proliferation, apoptosis, senescence, angiogenesis, adhesion and migration^65^. To the best of our knowledge, dysregulated expression of KLF14 is not reported in brain cancer so far. This study reported that the expression of KLF14 in brain tumours is down-regulated as compared to healthy controls suggesting its expression is necessary for the normal functioning of brain. The expression of KLF14 is also suppressed in other human cancers, supporting its tumor suppressive role^13^. This study further focuses on the expression of KLF14 in relation with metastatic status and tumor grade. Downregulation of KLF14 was observed in metastatic group, suggesting KLF14 down-regulation might have role in cancer metastasis. KLF14 expression variation was also observed in different brain tumour grades. In lower grade of brain tumours (I + II), its expression is higher than the advanced grade tumours (III + IV), suggesting the continuous reduction of KLF14 expression in brain tumours may lead towards the high-grade cancer. The present study also demonstrated the novel finding of lower expression of KLF14 in space occupying lesion (SOL) of brain compared to healthy controls; however, KLF14 expression in blood of brain cancer patients was lower than that observed in SOL patients.

Strong scientific evidence indicate the overexpression of TPD52 in many types of cancer^9,22,25,36,66^. However, no evidence was previously available delineating its expression in brain cancer. In the current study, TPD52 expression in brain cancer was found to be up regulated which agrees with the previous study^67^ that further strengthened the oncogenic role of TPD52. Furthermore, TPD52 expression increased with cancer grade and was also found to be associated with brain cancer primary metastasis. Previously, TPD52 expression up-regulation with cancer stage progression was reported in breast cancer^36^. Additionally, TPD52 expression was found to be lower in brain SOL than brain cancer but higher compared to healthy control. Increased levels of TPD52 in SOL of brain may be a key player that leads toward cancer. Taken together, TPD52 may be a potential biomarker and effective target to improve therapeutic strategies for better treatment of brain tumour.

A tumor suppressive role for miR-124 in different cancers including colorectal, hepatocellular and gastric carcinoma is well-established^68–70^. MiR-124 down-regulation in gliomas is also reported^26,34^. The current study showed reduced expression of miR-124 in whole blood of brain tumour patients. Expression of miR-124 is also down-regulated in different sub-types of brain cancer such as oligodendroglioma, astrocytoma and GBM^31–33^. Our findings broadened the knowledge on miR-124 contribution in brain cancer by demonstrating its decreased expression in secondary metastatic brain tumors. Xia et al.^29^ showed that restored miR-124 expression in GBM inhibited cancer invasiveness. Further, higher reduction of miR-124 in high-grade cancer than in lower-grade cancer was found in current study. Study of Wie et al.^26^ also reported down-regulation of miR-124 in all grades and pathologic types of gliomas. It is possible that reduced expression of miR-124 contributes to the transition from low to high-grade cancer e.g. GBM. Similarly, the down-regulated levels in SOL of brain relative to health control but up-regulated levels relative to brain cancer suggest miR-124 expression loss might be part of the transformation process of SOL into tumor. Our results opened a new avenue for elucidating the importance of miR-124 in normal functioning of brain and how loss of miR-124 contributes to brain malignancy.

Expression of PKCε was also investigated in brain tumours. Consistent with the notion that PKCε is upregulated in many human cancers e.g. prostate, lung and breast cancer^71^, it was found to be over-expressed in brain tumours as well. Our work is in agreement with the previous study in which it was found to be overexpressed in different types of brain tumours^38^. Extensive study has been focused to find out its expression in different clinical features of brain tumours. Elevated expression of PKCε leads toward the metastasis of brain tumours to different organs^38,72,73^. In this study, PKCε levels were observed to be lower in grade I + II compared to advanced grade III + IV brain tumour. This data supports that continuous upregulation of PKCε is associated with higher grade cancer, confirming PKCε as a key player in brain tumours. Further this study provides novel data for a role of PKCε in SOL of brain. It was observed to be upregulated in space occupying lesions (SOL) of brain compared to healthy control. PKCε expression was higher in cancer patients compared to SOL brain, again providing a gradient of increased levels from healthy brain to SOL and finally to brain cancer. Our results confirm the role of PKCε as an oncogene in brain tumours; PKCε poses a potential biomarker for this disease, which warrants verification in further studies. It may be an effective target to improve therapeutic strategies for better treatment of brain tumour.

Pathway analysis indicated the interaction of under-studied genes with major signalling cascades, including the Akt pathway and KRAS signalling. KLF14 negatively modulates the functioning of oncogenic KRAS^74^, whereas PKCε brings about activation of KRAS^75^. KLF14 is present downstream of PKCε and its down regulation in cancer supports cell proliferation and survival. In prostate cancer, elevated KLF14 expression promotes cancer survival by initiating mechanisms attenuating metabolic processes induced oxidative stress^17^. KLF14 also modulates activity of PLK4 and suppresses amplification of centrosomes^76^. However, KLF14 loss enhances PLK4 that leads to carcinogenicity^13^. TPD52 acts as a bridge between receptor tyrosine kinases and Akt and promotes pro-survival signalling through Akt pathways^66^. Contrary to TPD52, expression of miR-124 attenuates signalling through Akt and associated downstream pathways^10^. Differential expression analysis of these genes in brain cancers and their molecular interaction in cellular pathways helped us in gaining insight of possible crosstalk to these genes.

Previous work revealed the participation of PKCɛ in activating the GPCR coupled Ras/Raf pathway that facilitates growth of neuronal cells that are associated with memory^77^. A regulatory link of PKCε with STAT3 has also been established in prostate adenocarcinoma^78^. A recent study ascertains the activation of STAT3 via TPD52 in neuroblastoma^79^. Hence transcriptional activity of STAT3 is regulated by PKCε and TPD52 as well as Rho-kinases. PKCε involvement was also found in Rho signalling; specifically, PKCε mediated activation of Rho GTPase to facilitate metastasis in lung cancer^80^. Evidence from literature also indicated that ERK phosphorylation in Ras/Raf pathway is due to activation of downstream target of PKCε i.e. Rho GTPases^81,82^.

Involvement of PKCε in the Akt pathway was also revealed by our pathway analysis. PKCε is located upstream of TPD52 and both genes activate Akt signaling that promotes tumor proliferation and invasion. Phosphorylation of Akt at serine 473 induces its activation^83^. Akt regulates proliferation and cell cycle by targeting cyclin D1, p21, p53 and p27^84,85^. Forkhead box O (FOXO) is the transcription factors and serve as downstream targets of Akt (protein kinase B). Akt inhibits FOXO by phosphorylating it and hence promotes cell survival, growth, and proliferation. Similar to PI3K/Akt signaling, TPD52 and PKCε block the transcriptional activity of FOXOs (specifically FOXO1, 3, and 4), activate cyclin D and inactivate p27 (a negative regulator of cell cycle), leading to enhanced cellular proliferation^86^.

Studies indicate the role of miR-124 as a tumour suppressor gene which plays an important role in cell apoptosis. Evidence show that increased expression of miR-124 in cancer cells blocks proliferation by inhibiting the KRas pathway^69^. miR-124 also causes the inhibition of cyclin dependent kinase 4 (CDK4), an activator of a pro-survival transcription factor E2F1, thus promoting cell senescence and apoptosis^68^. Further the dysregulated expression of miR-124 leads toward the increased expression of SLUG. Its role is to bind with the promoter region of E-Cadherin that causes cell invasion^87^.

The outcomes of the current study highlighted the diagnostic potential of co-expression of KLF14, PKCε, TPD52 and miR-124 in brain cancer. Moreover, evaluation of these genes at the protein level will further validate their efficacy as blood-based biomarkers for the diagnosis and prognosis of brain cancers. Differential expression of these on a large cohort size of different subtypes of brain cancer can further unravel the subtype-specific diagnostic efficacy of these genes.

## Conclusions

In the current study, we investigated the co-expression profile of KLF14, TPD52, PKCε and miR-124 and found up-regulated expression of TPD52 and PKCε and down-regulated expression of KLF14 and miR-124 in peripheral blood of representative solid brain tumour and SOL patient samples. Dysregulation of these genes has been found to be associated with disease progression. These findings reveal the important role of these genes in brain tumour and SOL of brain, highlighting their role in brain functioning. Also, our results suggest their importance as a potential biomarker and therapeutic target for brain tumours. Further validation of co-expression of these genes in blood of brain cancer patients will be advantageous by providing less invasive means for early diagnosis of the disease.

## Data availability

All the data is contained in the manuscript.
